# A Phylogenetic Comparative Study of Bantu Kinship Terminology Finds Limited Support for Its Co-Evolution with Social Organisation

**DOI:** 10.1371/journal.pone.0147920

**Published:** 2016-03-23

**Authors:** Myrtille Guillon, Ruth Mace

**Affiliations:** Human Evolutionary Ecology Group, Department of Anthropology, University College London, London, United Kingdom; British Columbia Centre for Excellence in HIV/AIDS, CANADA

## Abstract

The classification of kin into structured groups is a diverse phenomenon which is ubiquitous in human culture. For populations which are organized into large agropastoral groupings of sedentary residence but not governed within the context of a centralised state, such as our study sample of 83 historical Bantu-speaking groups of sub-Saharan Africa, cultural kinship norms guide all aspects of everyday life and social organization. Such rules operate in part through the use of differing terminological referential systems of familial organization. Although the cross-cultural study of kinship terminology was foundational in Anthropology, few modern studies have made use of statistical advances to further our sparse understanding of the structuring and diversification of terminological systems of kinship over time. In this study we use Bayesian Markov Chain Monte Carlo methods of phylogenetic comparison to investigate the evolution of Bantu kinship terminology and reconstruct the ancestral state and diversification of cousin terminology in this family of sub-Saharan ethnolinguistic groups. Using a phylogenetic tree of Bantu languages, we then test the prominent hypothesis that structured variation in systems of cousin terminology has co-evolved alongside adaptive change in patterns of descent organization, as well as rules of residence. We find limited support for this hypothesis, and argue that the shaping of systems of kinship terminology is a multifactorial process, concluding with possible avenues of future research.

## Introduction

### Kinship and Anthropology

Human kinship, consisting of networks which group individuals as relatives, is a feature of social organization which is globally recognized, and given importance to [1]. Kinship is not to be conflated with biological relatedness, as it has long been acknowledged that different cultures ascribe variable social importance to different kin types, in a way which does not necessarily reflect degrees of genetic similarity. In the words of Wagner, “the essence of kinship is interpretation of genealogy, rather than genealogy itself” [2]. The cross-cultural study of this phenomenon has been of interest since the foundation of Anthropology in the nineteenth century [3, 4]. Following his ethnographic study of the matrilineal Iroquois, Morgan realized that the Western approach to kinship organization was not universal, and began collecting ethnographic data to investigate this variation. He theorized that the cultural diversity of kinship systems observed throughout the world was explained by a process of ‘unilinear evolutionism’, with different groups falling along a single scale of social evolution that ranged from ‘primitive’ to ‘civilised’ [3, 5]. Influenced by his admiration for the field of philology, Morgan utilized data on kinship terminology—which are the terms used to refer to and describe one’s kin group members—and suggested that these could provide insight into the diversity of human social structure [3]. He began the gradual process of classifying kin terminologies into typologies based on common features, separating those which distinguished between lineal kin (direct ancestors and descendants) and collateral kin (siblings and cousins) and those that do not. He identified groups which make the distinction as using descriptive terminology (referring to one or few relatives), while those that do not were said to use a more primitive classificatory terminology, which grouped together a large number of relatives into one category [3]. Although these early, and somewhat Lamarckian, evolutionary theoretical frameworks have long been discredited [6], that is not to say that evolutionary forces have had no influence in shaping the kinship system diversity which is found throughout the world. We now know that evolution does not operate in a unilineal fashion, and that there are no necessary pathways to ‘progress’, but rather that cultural variation in social organization is caused by adaptation to differing environmental pressures, each of equal appropriateness according to local constraints. After all, cultural concepts of kinship underlie the organization of activities which are vital to evolutionary success; such as the finding of a mate, the caring for offspring and the production of food [7]. Given that these crucial behaviours are constrained by the structural organization of kinship, evolutionary theory would predict strong selective pressure influencing such structure, and favouring the survival over time of kinship systems that produce adaptive structuring. In accordance with this hypothesis, it has been observed that although there is extensive variation in kinship terminology across the world, there are nowhere near as many forms as could logically be thought of, and there is patterning in the variation found [8].

Although Morgan was working within an invalid framework, he made a vital contribution to the study of kinship terminology diversity, by beginning the classification of systems for a comparative approach, and suggesting that these groupings were informative for the categorisation of variation found in other aspects of social structuring [3]. In a famous study published in 1909, Alfred Kroeber reasoned, “It is apparent that what we should try to deal with is not the hundreds or thousands of slightly varying relationships that are expressed or can be expressed by the various languages of man, but the principles or categories of relationship which underlie these” [9]. Kroeber identified eight such categories of distinction; generational, differentiation between collateral and lineal relationship, within-generation age, sex of the relative, sex of the speaker, sex of the connecting relative (known as ‘bifurcation’), the differentiation of consanguineal and affinal kin and the differentiation between living and dead kin. He concluded his seminal paper by suggesting that kinship terminology was primarily a psychological and linguistic phenomenon, and as such should not be used for sociological interpretation without extreme caution [9]. By the 1950s, most anthropologists agreed that kinship terminology was shaped by local social structure, at least to some degree ([10], See S1 File). An influential view of the time is that of Radcliffe-Brown, who was a student of W.H.R.Rivers, and argued that the function of kin terms was to act as a guide for interpersonal conduct, with different social duties or obligations being ascribed to terminologically-defined kin categories [11].

**Fig 1:**
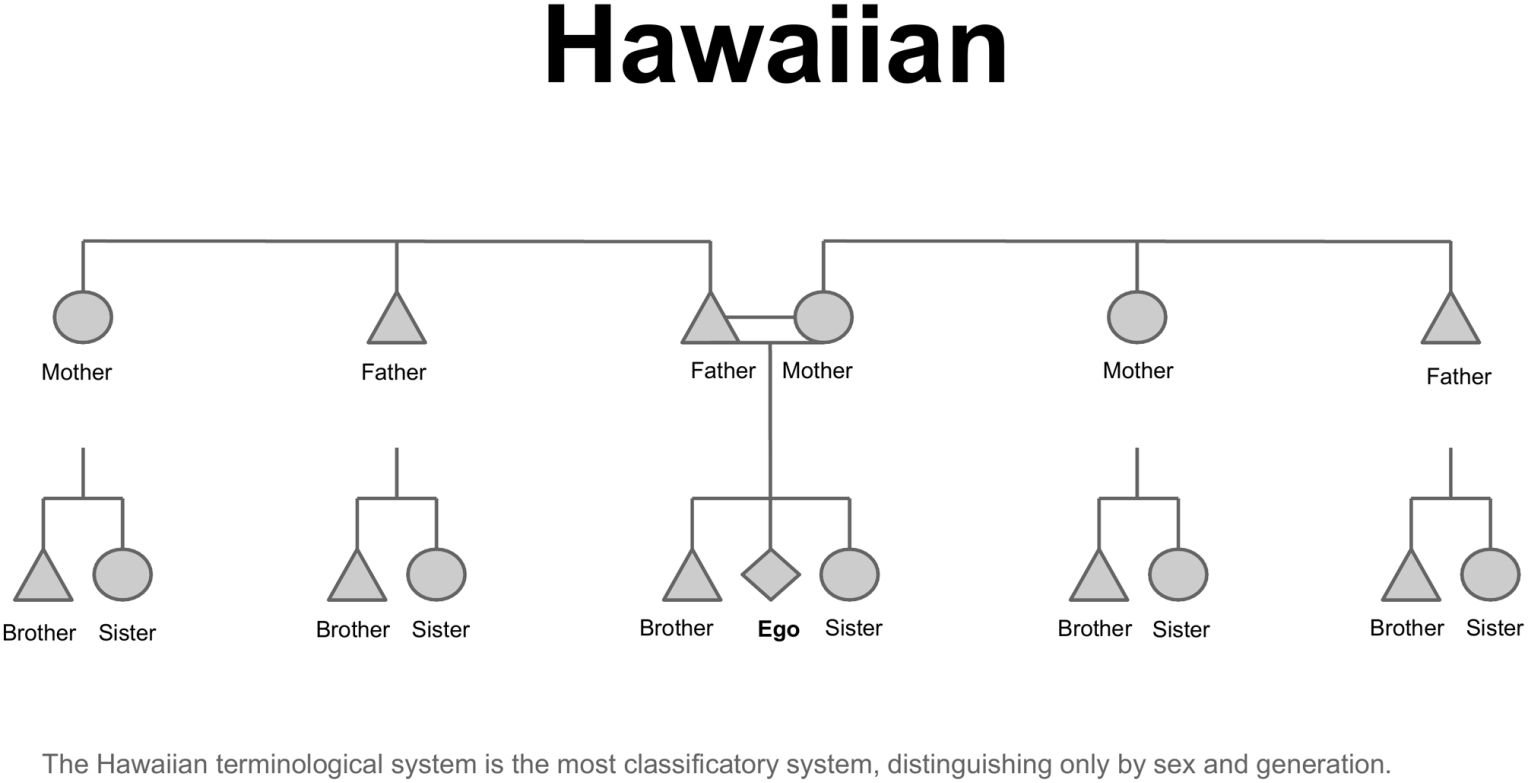
Classic typologies of kinship terminology. The six typologies created by Murdock, and how kin is referred to in relation to ego in each system.

Despite this limited consensus, there was no theory for the social determination of kinship terminology which was unified nor widely accepted [12], although several factors of influence had been suggested throughout the preceding decades. Customs of preferential marriage were often argued to influence certain structural aspects of kin term variation, such as the suggestion that sororal polygyny might minimize the importance of the collaterality distinction, and encourage terminology of the bifurcate merging type [13, 14]. The other main type of social factor which was often suggested was that of the composition of local kin groups, such as the presence of moieties [15], or matrilineal versus patrilineal groupings [16, 17]. It is around this time that Murdock published ‘Social Structure’, which represents a massive forward leap in the cross-cultural study of kinship. Murdock combined a synthesis of findings from psychology, sociology and anthropology with his extensive collection of global cultural data to advance his own theory of human social evolution [18]. Building on the work of Spier, who had suggested the use of cousin terminology typologies for cross-cultural comparison [19]; Murdock created six typologies which encompass the global variation of cousin terminology [18]. These typologies are known as Hawaiian, Eskimo, Iroquois, Omaha, Crow and Sudanese ([18], see Fig 1).

Murdock also refined Kroeber and Lowie’s concept of categories of distinction. He reasoned that generation, sex, affinity, collaterality, bifurcation and polarity were the six main distinctions, as the linguistic non-recognition of either one of these produces classificatory, as opposed to descriptive, terms [18]. Within-generation age, speaker’s sex and the differentiation between living or dead relatives are subsidiary categories, which make classificatory terms less inclusive and descriptive terms more specific when they are linguistically recognised [18]. Together, these categories represent all the principles used by humans to linguistically delineate between kin, and whether they are recognised or not is likely to give insight into what is driving the structural variation of kinship terminology [18]. Murdock formulated over thirty hypotheses about the relationship of kinship terminology and social structure, which he tested cross-culturally using a bivariate statistical method, and a combination of Lowie’s four typologies, categories of fundamental distinction and the six cousin typologies. Murdock found that kinship terminology structure was primarily determined by three different aspects of social organization which can have opposing influences; rules of descent, group norms concerning marriage and rules of residence after marriage—in order of respective importance [18]. Many of his findings were replicated in the only cross-cultural study which has attempted to do so, using a multivariate statistical method which allows for the graphical visualization of associations between categorical variables, known as optimal scaling [20].

Since the 1970s, a variety of factors have complicated the anthropological study of kinship, and led to the falling out of favour of its cross-cultural study. One historical complication of major relevance here is that of Galton’s problem. Upon the presentation of Tylor’s comparative research on the diversity of kinship systems found across cultures, Galton pointed out that closely related human populations could not be treated as being statistically independent from one another, as they may share traits through common inheritance from a recent ancestor [15]. Due to this fact, comparative studies in anthropology have not traditionally been able to reliably distinguish between genuine instances of parallel cultural evolution, and those groups which resemble each other because of shared descent [21].

### Modern approaches to the cross-cultural study of kinship

Modern evolutionary anthropology does not posit that cultural evolution is unilinear, or even that there are any necessary evolutionary pathways for cultural change. Rather, it seeks to understand human behavioural variation as a maximisation of inclusive fitness according to varying environmental constraints, with kinship organization being a crucial influence [22, 23]. The sub-field of human behavioural ecology in particular, highlights the fact that evolution is not progressive, as it sees cultural diversity in social organization being shaped through adaptation to differing environmental and social pressures, each form being of equal validity according to local constraints. Kinship organization displays much diversity across cultures, but a small number of core principles underpin this range, and allow the categorisation of all human kinship into a manageable number of groupings. First, the principle of descent refers to the way in which groups trace their ancestry and determines group membership of kin [1]. Descent may be unilineal, and trace ancestry exclusively through the maternal line (matrilineality), exclusively through the paternal line (patrilineality) or equally through both lines (bilaterality) [18]. Alternatively, descent can be ambilineal, meaning that individuals can align themselves with either paternal or maternal kin depending on their choice [24]. Second are rules of residence after marriage; these may be matrilocal (the couple resides with the wife’s kin), patrilocal (the couples resides with the husband’s kin), neolocal (the couple resides in a nuclear family unit away from either kin group) or duolocal (husband and wife live apart, each with their respective kin group) [18]. A third crucial principle is that of group-level marriage norms, as human populations may be either predominantly monogamous (where one man is married to only one woman), polygynous (where one man has several wives) or polyandrous (where one woman has several husbands) [18]. These categories can of course be broken down further by considering for example; rules of endogamy and exogamy in kin groups, preferential marriage customs (eg cross-cousin marriage, sororal polygyny), or rules of exchange at marriage (dowry or bridewealth). The field of human behavioural ecology has in part focused on how these principles interact together and with ecological pressures, mainly through individual-level analyses, but any attempt at testing these hypotheses cross-culturally would be hampered by Galton’s problem.

The emergence of cultural phylogenetics, has represented a significant advance in the cross-cultural study of kinship. Through the adaptation of phylogenetic statistical methods developed in evolutionary biology for the comparative study of hierarchically related species, it has become possible to test hypotheses about human cultural evolution while accounting for Galton’s problem [21, 25]. As suggested by Mace and Pagel in their seminal article, integrating phylogeny into comparative analyses allows for the detection of independent instances of cultural change, unlike previous attempts at solving the problem [21]. This crucial development has resulted in the flourishing of a new field, in which testing hypotheses about the evolution of kinship diversity has had an important place. Holden and Mace [26, 25] investigated the long suggested association between cattle-keeping and patrilineal descent [27], and found that in Bantu-speaking populations the adoption of cows as a means of subsistence usually resulted in a switch to patrilineality, and that once cattle-keeping was present in a group it became very unlikely to become matrilineal. Fortunato and colleagues tested the widely accepted hypothesis that bridewealth systems of marriage exchange were ancestral to systems of dowry [28], and found the opposite to be true in Indo-European groups. Jordan [29] investigated the ancestral Austronesian rules of residence after marriage, showing that the earliest speakers of this family of languages were matrilocal. Fortunato and Jordan [30] reconstructed the past forms of marital residence, and the rates of change between these different forms of residence rules in Austronesian and Indo-European groups.

Studies in this vein have not only revisited kinship but enlightened the study of such varied topics as the evolution of political complexity [31] and how it has interacted with processes of social stratification [32], of the relationship between sex ratio at birth and markers of life history pace [25], the associations between sexual dimorphism and subsistence strategies [33] and the co-evolution of pastoralism and lactose tolerance [34]. But in order to reliably test these hypotheses, the first step is to construct accurate phylogenetic trees, the most appropriate being the phylogenies of language families in the case of cultural evolution [21]. These linguistic phylogenies act as an underlying model of population history, by statistically inputting the historical relatedness of groups into the analysis, based on the relationship between comparative elements of core lexical vocabulary [35]. In the past fifteen years these methods have produced phylogenies for at least three major language families; Austronesian [36, 37], Indo-European [38] and Bantu [39, 40]. These phylogenies have been considered to be realistic models of population history by archaeologists, linguists and historians [41], and have permitted to formally and independently test between different scenarios of population expansion [36, 38, 40, 42].

The transition from a hunting and gathering lifestyle, to that of agricultural food production, characterised by sedentarisation and an increased population size and density, would have necessitated important adjustments in kinship organization [43]. For those small-scale agricultural societies which are not ordered at a state level, such as our study sample of historical Bantu populations, the majority of everyday social activities and the structuring of cooperative networks are regulated through the functional organization of kin [1, 43]. In particular, if we take on Radcliffe-Brown’s assertion that terminological variation in kinship has the function of delimiting kin categories in the context of social obligations, understanding this transitional restructuring would inform us about the evolution of kinship as the earliest institution of human cooperative behaviour. One study has employed phylogenetic comparative methods to investigate the evolution of kinship terminology, reconstructing the ancestral state and modelling the semantic evolution of Austronesian sibling terminology [7]. However, no study has used these methods to explore the relationship between variation in kinship terminology and principles of social structuring, an attempt which will be made in the present study.

### Study population

The Bantu languages represent one of the biggest linguistic families in the world, numbering over five hundred and spanning across the whole of sub-Saharan Africa [40]. The origin of this group has been traced to the Benue valley of eastern Nigeria, around 5000 years ago [44]. Archaeological, linguistic and genetic approaches have all indicated that Bantu speakers dispersed and expanded throughout sub-Saharan Africa, a process which has been dated as beginning between 3000 and 5000 years ago, departing from the Nigerian-Cameroon border area [45–47]. Today, it is in this geographical area that the languages most closely related to Narrow Bantu are found [48]. It has been hypothesized that the Bantu population expansion, like several others throughout the globe, was spurred by the spread of agriculture [49]. The adoption of a food-producing strategy would have allowed Bantu populations to thrive and expand into areas occupied by hunter-gatherers, displacing or assimilating these groups in the process [49]. Proto-Bantu groups are thought to have been largely dependent on hunting, gathering and the cultivation of yams, and that the eventual adoption of cereal crops coupled with metallurgy is what fuelled the expansion of Bantu speakers [50]. Vansina [50] suggests that Proto-Bantu society had a bilateral descent and bi-local residence system that was adaptive for expanding populations. He argues, based on linguistic reconstruction, such as the proto-Bantu word for ‘house’ being gender neutral, that residence was ambilocal [51]. Hunting required co-operation and mobility, and would be best served by males having a choice about their residence rather than being constrained by unilocality [50]. Only in the eighteenth or nineteenth century, due to increased wealth and the disorder faced by some Bantu-speaking people, did unilineal descent and residence patterns begin to emerge, it is suggested [50]. Another theory proposes a unilineal descent and unilocal residence system in the ancestral Bantu population [52]. Hage and Marck argue, based on the linguistic reconstruction of kin terms, that the early Bantu speakers were in fact matrilocal and matriline, and dispute the gender neutrality of the proto-word for ‘house’.

The study of Bantu languages has interested scholars from a variety of disciplines, and a number of attempts have been made to phylogenetically infer the relatedness structure of these ethnolinguistic groups. Most notably, Bastin and colleagues used distance methods to assess levels of similarity between comparative wordlists of 542 languages [53]. The lexical data collected for this study are a near-complete sample of Bantu languages, and use meanings which are relatively insensitive to borrowing. The data quality is therefore exceptionally good, but the study could not yield any conclusive inference about the historical relationships between languages, due to the nature of the phylogenetic method used. Distance-based techniques, like lexicostatistics, cannot differentiate between cultural retentions and cultural innovations, and they inherently assume a constant rate of evolutionary change, which is problematic when trying to make assessments of historical relationships [54, 55]. In a series of studies, Holden and colleagues used a sub-sample of these data published in the Bastin et al study, selected for the purposes of cross-cultural analysis, and applied character-based methods of phylogenetic inference [39, 56, 57]. Character-based methods, such as maximum parsimony, maximum likelihood and Bayesian Markov chain Monte Carlo, are a significant improvement, as they use cognate change for phylogenetic inference, rather than overall distance [58]. Similarly, Rexova et al combined a sub-sample of the lexical data from Bastin et al with grammatical data, and applied character-based techniques of inference [58]. Although these studies made useful findings, they are limited by some issues. In both cases, the methods used constrained the sample size dramatically, meaning that the majority of the lexical data from the Bastin et al study could not be used, and we know that a relatively small sample size can reduce the performance of phylogenetic methods [59]. Also, both of these sub-samples contained relatively few groups from the Northwest and rainforest, groups which are key to understanding the early diversification and expansion process due to their proximity to the Bantu homeland.

Taking on the lessons learned from these previous studies, a new Bantu phylogeny was published [40]. This study has produced the most accurate Bantu tree to date, through a number of key improvements in design. First, the phylogeny is based on the full sample of data available from Bastin and colleagues’ study, meaning that it has a large sample size and even linguistic data sampling across the whole of the Bantu-speaking area, including those near the homeland. Second, the phylogeny was inferred using a method which allows for linguistic rates of change to vary, because it has long been suspected, and recently been shown empirically, that language does not evolve at a single rate [60, 61, 62]. Third, the study used Bayesian Markov Chain Monte Carlo methods of inference to produce a sample of five hundred phylogenetic trees [40]. These methods are a dramatic improvement over other character-based methods because they explicitly incorporate uncertainty in tree topology, as no one tree is likely to represent a true historical model [25]. The sample of trees produced in this study will be used here in our attempt to apply phylogenetic comparative methods to the co-evolution of kinship terminology and social structure.

### Hypotheses and Study Design

The creation of kinship terminology typologies has been useful in identifying the broad structural differences between types, and has led to the observation that societies in opposite parts of the world have independently come up with comparable cultural concepts of kinship organization, as we would theoretically expect from a standpoint of modern evolutionary theory. Although there has never been consensus regarding the causality which underlies the shaping of the different typologies, there are suggested associations which have repeatedly been found and become partially accepted through time. These following associations consist of the linking of a terminology typology with a system of descent, a likely evolutionary association as these systems represent distinct strategies for the organization of kin into groups of ancestry, and different grouping strategies result in distinct roles for the same genealogical kin across descent systems, which may be reflected in the structuring of kinship terminological diversity. Hawaiian systems are thought to be mostly found in groups which trace their descent ambilineally, as this type of descent appears to require a more flexible—hence simplified—terminology [63]. Iroquois type terminology is thought to be associated with groups which practice unilineal descent organization, as this would require the bifurcate emphasis which is characteristic of these terminologies [64]. Omaha systems, with their descriptive terms for paternal kin contrasting classificatory terms for maternal kin, are thought to be associated with groups which have a strong patrilineal influence in their social organization [64]. Inversely, Crow systems are thought to be found in groups with a strong matrilineal influence on overall social organization [64]. As with any cultural phylogenetics study, before proceeding to the testing of co-evolutionary hypotheses, it is crucially important to visualize the data on the phylogeny, as this key step should inform and guide further investigation. Here, we present a graphical representation of the reconstructed evolution of Bantu kinship terminology across time, complete with ancestral terminological states at the root and historical nodes of the tree.

In this series of analyses, we then use cultural phylogenetic methods to formally test these long-held hypotheses about the co-evolution of kinship terminology diversity and systems of descent, and make use of the recent improvements in Bantu phylogeny to test these hypotheses in sub-Saharan African ethnolinguistic groups. Our research questions are:

1 Has Iroquois terminology co-evolved with unilineal descent among Bantu groups?
2 Has Hawaiian terminology co-evolved with ambilineal descent among the Bantu?
3 Has Omaha terminology co-evolved with patrilineal descent in Bantu groups?
4 Has Crow terminology co-evolved with matrilineal descent in the Bantu?

We then test the hypothesis that kinship terminology has co-evolved with local rules of residence, asking:

5 Has Iroquois terminology co-evolved with rules of unilocal residence among the Bantu?
6 Has Hawaiian terminology co-evolved with rules of ambilocal residence in Bantu groups?
7 Has Omaha terminology co-evolved with rules of patrilocal residence among the Bantu?
8 Has Crow terminology co-evolved with rules of matrilocal residence among the Bantu?

## Methods

### Reconstructed evolution of Bantu kinship terminology across time

Ancestral state reconstructions of kinship terminology were carried out using the Multistate feature of BayesTraits (available from http://www.evolution.reading.ac.uk/BayesTraitsV2Beta.html). The full sample of 500 Bantu phylogenies ([45], refer here for the tree-building methodological details) and all groups which could be reliably matched between the linguistic populations present on the tree and Bantu populations from the Ethnographic Atlas were used (N = 120). Refer to appendix of [45] for ethnolinguistic matching list. The results were then summarised into a full consensus tree using the program BayesTrees, and coded for graphical representation using the Ape package in R. All analyses were run using Bayesian Markov Chain Monte Carlo methods.

In order to find the posterior probability distributions of states at ancestral nodes across the phylogenies an RJ MCMC analysis was run with the additional use of the addNode command in Bayes Traits, which reports the posterior probability of trait states at each internal node on the phylogenetic tree [65]. The ancestral state reported for each node of the tree is the combined posterior probability of each state at that node with the posterior probability that the node itself exists in the tree, and is plotted on the consensus tree.

### Co-evolutionary hypotheses testing

To test these hypotheses, we are using the Ethnographic Atlas [66, 67], which contains global data on cousin terminology (variable 27), categorising societies under one of the six Murdock typologies, as well as global data on descent practices (variable 43), and rules of residence after marriage (variable 11). In order to employ phylogenetic comparative methods, we use a recently published Bayesian sample of 500 phylogenetic trees, informing our analysis of the structure of relatedness between 542 Bantu languages of sub-Saharan Africa [40]. The sample is therefore restricted to Bantu ethnolinguistic groups which contain data for cousin terminology in the Ethnographic Atlas, and could reliably be matched to linguistic data used to infer the phylogenetic trees [53], resulting in a total of 83 populations. According to Murdock’s Ethnographic Atlas, across our Bantu sample, the variation found is as follows: 15 groups use Hawaiian terminology, 51 groups use Iroquois terminology, 9 groups use Omaha terminology, 5 groups use Crow terminology, 2 groups use Sudanese terminology and 1 group is classified as ‘mixed’. All analyses were carried out in the Discrete implementation of BayesTraits, using Bayesian Markov Chain Monte Carlo methods with RJHP (exponential at interval 0 to 4). The analysis produces two models of cultural evolution, one in which the two binary cultural traits evolve independently along the phylogenetic branches (independent change model), and one in which the probability of change in one trait is dependent upon the state of the other trait (dependent change model) [68]. The method produces a set of parameter values (representing the rates of character change) which make the observed data most likely, given the model of population history [68]. The likelihood of the two models—which indicate how well the model fits the data—can then be compared, if the dependent model is a statistically significant improvement over the independent model, the results provide support for co-evolution between the traits of interest [68].

## Results

**Fig 2:**
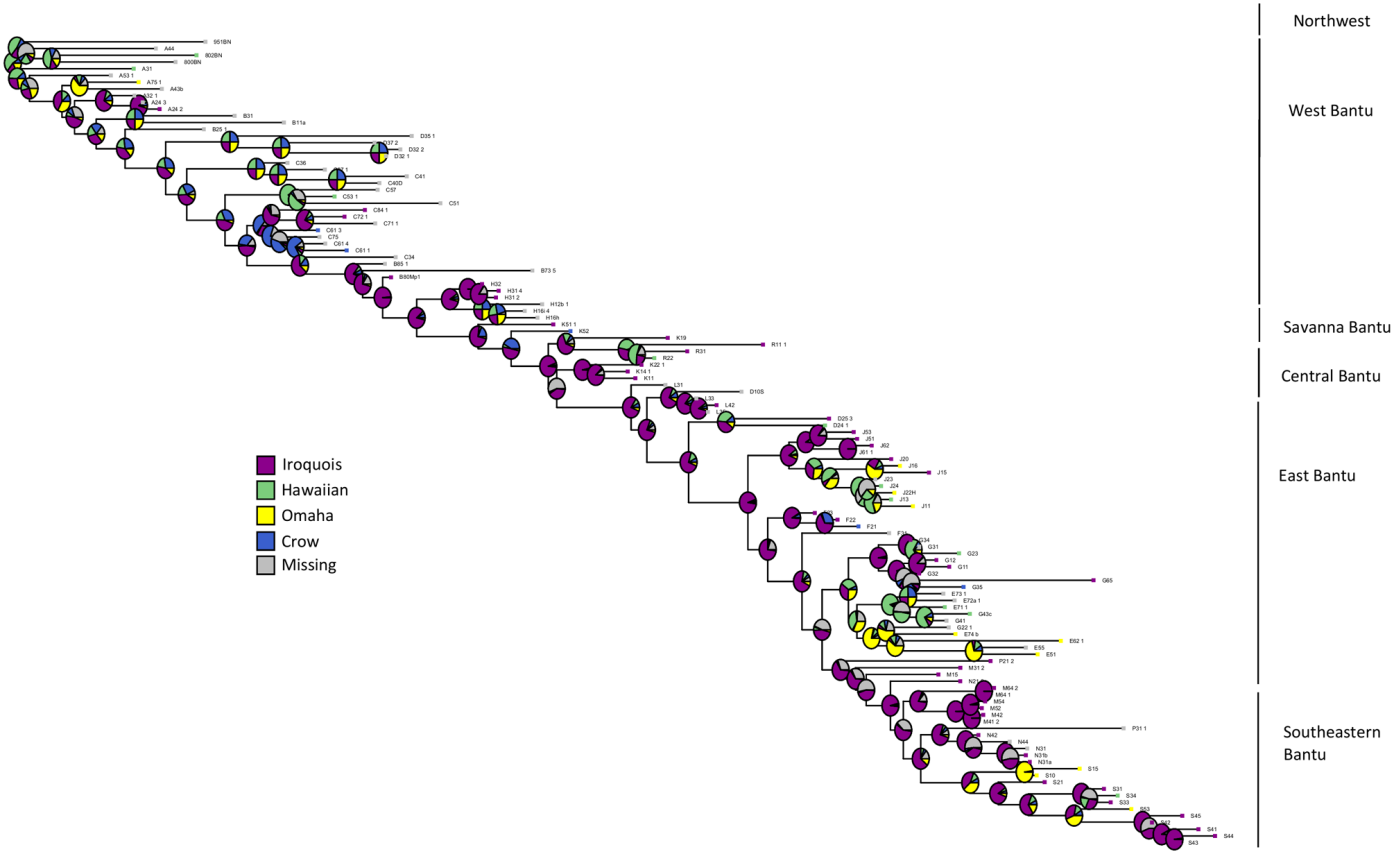
Ancestral state reconstruction of Bantu kinship terminology. Bantu phylogenetic consensus tree with a state reconstruction of kinship terminological systems, with the mean probability of state illustrated in pie charts at each node of the tree.

We find only very weak support for the commonly held assumption that systems of kinship terminology, as traditionally classified, are co-evolving with patterns of descent organization. Mapping out the within-Bantu variation in terminological systems (Fig 2) helps us see that there is some differential clustering across the phylogenetic tree, with the northwest and equatorial West Bantu groups showing more homogeneity and a prevalence of Iroquois-type systems, while in contrast, the southeast East Bantu, central Bantu and Savannah West Bantu show more heterogeneity and perhaps some specialisation into the more strictly patrilineal unilineal system, Omaha, or the mirror matrilineal system, Crow. But when we test this hypothesis of association, that the variation of terminological systems may be associated with local rules of descent organization, we find either no evidence or only weak evidence for co-evolutionary relationships (Tables 1, 2 and 3).

**Table 1:** Bayes Factor Interpretation, adapted from [69].

Log Bayes Factor	Interpretation
<2	Weak evidence
>2	Positive evidence
5–10	Strong evidence
>10	Very strong evidence

Table for interpretation of Bayes Factor analysis, adapted from [69]. Log BF = 2(log[harmonic mean(complex model)])–log [harmonic mean(simple model)].

**Table 2:** Kinship Terminology and Descent Rule Co-Evolution.

Association	Independent Model	Dependent Model	Bayes Factor
Iroquois + Unilineal	-86.384	-85.542	0.358 (±0.009)
Hawaiian + Ambilineal	-53.869	-46.825	3.048 (±0.011)
Omaha + Patrilineal	-95.195	-93.244	0.841 (±0.007)
Crow + Matrilineal	-74.832	-74.346	0.215 (±0.003)

The Likelihoods of the independent and dependent model for four cases of the possible co-evolution of kinship terminological systems and rules of descent, tested using Discrete. Statistcial support for the dependent model being significanty more likely than the independent model is measured by a Bayes Factor.

**Table 3:** Kinship Terminology and Residential Rule Co-Evolution.

Association	Independent Model	Dependent Model	Bayes Factor
Iroquois + Unilocal	-79.699	-79.258	0.191 (±0.016)
Hawaiian + Ambilocal	-68.622	-68.127	0.215 (±0.003)
Omaha + Patrilocal	-91.157	-87.289	1.680 (±0.002)
Crow + Matrilocal	-71.248	-70.324	0.401 (±0.004)

The likelihood of the dependent and independent models for four cases of the possible co-evolution of kinship terminological systems and rules of residence, tested using Discrete. Statistical support for the dependent model being significantly more likely than the independent model is measured by a Bayes Factor.

The strongest association found was between Hawaiian-like terminological systems and ambilineal systems of descent. This could represent an older, Proto-Bantu specialisation into flexible systems of kinship organization and communication upon the African agricultural transition, whereby groups could easily become larger or assimilate groups of hunter-gatherers without encountering organizational issues in managing people and their duties within a hierarchical class system.

In contrast, when testing the relationship between kinship terminological systems and rules of residence after marriage, the strongest association we find is that between Omaha kinship terminology and patrilocal rules of residence after marriage (Table 3). This result that most language grouping do not show the predicted associations, and those that do are not the same for descent and for residence is surprising, as rules of descent and residence tend to be very closely interlinked.

At the root of the Bantu tree, we find strongest support for a Hawaiian ancestral state of kinship terminological organization, although other states are also possible. Of the varying hypotheses about what form of social organisation is at the root of the Bantu tree, this suggest support for Hage’s hypothesis that it is bilateral, but there is also some support for Iroqouis at the root. If Hawaiian is a language system flexible enough to have co-evolved in any lineal system, this is potentially compatible with either Vansina’s idea that it is matrilineal, or patrilineal, as Opie et al [70] suggest using the same tree sample and a similar methodology to that used here. However, our general finding that the correlation is low between terminology and social organisation suggests caution in attributing ancestral social structures on the grounds of Murdoch’s kinship terminology only.

## Discussion

Iroquois-like systems are not only the most common across the Bantu tree, but also widespread across the whole of the Sub-Saharan African continent. Iroquois terminology is more flexible than the matrilineal Crow and patrilineal Omaha systems. Iroquois systems are strongly associated with unilineal descent in Africa, used by both predominantly matrilineal and predominantly patrilineal groups. But there is no evidence from this study that unilineality and Iroquois co-evolve. It may be that Iroquois and unilineality are both so common in the Bantu tree that there is not enough variation in the tree to show co-evolution.

Generally the speed of evolution of descent may be faster than that of linguistic change in kinship terminologies. This was originally proposed by Murdock [18], with the idea that residence rules were first to change, triggering changes in descent, and followed significantly later by changes in kinship terminology. Certainly Currie et al [40] suggest the Bantu moved in and out of the forest on many occasions and Opie et al [70] show how kinship changed many times over the course of 6000 years, and Currie and Mace [71] show that the rate of evolution in kinship terminology is a little slower than other variables relating to social organisation. The costs and benefits of changing social organisation are lower than linguistic change, then language thus not necessarily tracking ecological and social change on similar time scales.

As Murdock first pointed out in Social Structure [18], the reconstruction of these evolutionary dynamics is being muddied by a problem of multifactorialism, whereby several very closely associated variables are influencing the shaping of the terminological system, but with different, and sometimes opposite influences (eg. sororal vs non-sororal polygyny). It is possible that Bantu populations adopted unilineal descent during the agricultural expansion, with certain groups further specializing in unilineal descent whilst adopting Crow or Omaha systems, but this hypothesis needs to be tested on a larger scale, perhaps across different family trees. Indeed, although Iroquois-like systems are strongly associated with unilineality in Africa and North America, they seem associated with bilateral cross-cousin marriage in Australian hunter-gatherers, such as the Kariera system, and perhaps also in South American native populations. This observation begs the cross-cultural question of whether Iroquois like systems evolved as a response to the spread of unilineal descent, or perhaps instead with practices of cross-cousin marriage in kinship groups of bilateral descent (such as ambilineal systems). This and our results also raise the broader question of whether Murdock’s original classification indeed captures the important aspects of social organisation that he supposed.

Despite the active agency of humans in shaping their history and cultural institutions, and the dramatically differing environmental conditions under which human populations have thrived, human cultural evolution can be studied and modelled according to general principles of neo-Darwinian evolutionary theory and behavioural ecology [72, 73]. A recently published study showed that the evolution of cultural traits related to social structuring occurred at comparable rates in speakers of the Bantu language family to populations of island Southeast Asia and the Pacific [71]. Although humans display very high behavioural flexibility relative to other species, and have culturally adapted to help buffer environmental risk, we are still subject to the general energetic and environmental constraints that all species face [73]. The Bantu represent a remarkably cohesive and closely related linguistic group, and as sub-Saharan Africa is highly ecologically diverse, we can expect vast differences in the cultural adaptation over time of groups which inhabit the Ituri rainforest, the African Great Lake area, and the savannah or desert, as the expansion took place. Kinship organization is a key aspect of human culture, bearing massive influence on the survival of small-scale populations. Amongst the historical Bantu chiefdoms that make up the sample of this study, kinship rules and social law were one and the same. In fact, kinship laws were so well respected that they have been found to be reflected in the genetic patterning of sub-Saharan African populations, with cultural rules showing to be a good predictor for explaining genetic variation across clan groups [74].

In the transition to farming, populations who were moving towards a more agricultural and sedentary lifestyle, but were not organized at a state level. Having a large family and following became synonymous with the accumulation of local power and dominance status [43]. Unlike the hunting and gathering groups native to the African continent, Bantu ethnolinguistic groups are not socially egalitarian, but rather represent the cultural adoption and spread of more socially stratified communities, and the competitive rise of African kingdoms and nation states, such as the Bunyoro or Baganda empires of the past, in present day Uganda. As such, we can look to these populations as representing a transition in human social organization. With the introduction of intense stratification, likely came the reshaping of rules of human cooperation, a behavioural aspect of human groups thought key to our adaptive spread and success. Although much of the evolution of human cooperative behaviour remains theoretically challenging to model, it has become clear that dominance and prestige opportunities are closely associated with the cultural adoption and spread of human rules of cooperation and punishment [75].

Many of these practices were interrupted and reformed by the influence of European colonial forces, making it difficult to reconstruct an accurate portrayal of native Bantu cultural evolution using historical sources alone. Nonetheless, phylogenetic comparative methods open up the possibility for the 'virtual archaeology' of cultural components which would be impossible to accurately model using more traditional, non-computational, anthropological or historical methods. We can therefore hope that these methods can help gradually disentangle these complicating factors, with the aim of reconstructing relatively objective models of African cultural evolution, and how these relationships compare across different language families.

## Supporting Information

S1 File Additional Detail. (DOCX)
