# Correction: A Phylogenetic Comparative Study of Bantu Kinship Terminology Finds Limited Support for Its Co-Evolution with Social Organisation

**DOI:** 10.1371/journal.pone.0155170

**Published:** 2016-05-03

**Authors:** The PLOS ONE Staff

Fig 1, “Classic typologies of kinship terminology,” is missing several images. Please see S1 Fig for the corrected Fig 1. The publisher apologizes for the error.

## Supporting Information

S1 Fig Classic typologies of kinship terminology. The six typologies created by Murdock, and how kin is referred to in relation to ego in each system.
(PDF)
