# Supplementary material for: A Phylogenetic Comparative Study of Bantu Kinship Terminology Finds Limited Support for Its Co-Evolution with Social Organisation
Source: PLoS One. 2016 Mar 23;11(3):e0147920. doi: 10.1371/journal.pone.0147920 (PMC4805278; doi:10.1371/journal.pone.0147920)
Supplement: S1 File — (DOCX) [file pone.0147920.s001.docx]

**Supporting Information**

**S1 File. Additional Detail.**

**The works of Rivers and Lowie**

One of Kroeber’s contemporaries, W.H.R. Rivers, espoused a strong ideological opposition to this rejection of kinship terminology as a reliable indicator of social structuring. In his book ‘Kinship and Social Organization’, Rivers set out to show that local features of social organization were a necessary influence in understanding kinship terminology variation, and could not be understood purely through linguistic analysis [1]. Building on this previous work, Lowie established the ninth fundamental category of distinction known as polarity, through the ethnographic observation that pairs of kin types sometimes use distinct terms to refer to one another - eg grandparent and grandchild, and sometimes use the same single term - eg cousin [2]. Lowie classified terminological structural variation into four typologies; generational (distinguishing neither the sex of the connecting relative nor whether kin are lineal or collateral), bifurcate merging (distinguishing between kin connected to ego through males or females but merging lineal and collateral kin), bifurcate collateral (distinguishing both the sex of the connecting relative and lineal and collateral kin) and lineal (distinguishing between lineal and collateral kin, but not the sex of the connecting relative) [2].

**Reasons for the decline in the anthropological study of kinship**

First, the field has been widely influenced by postmodernist philosophy, raising concerns about the validity and objectivity of comparison, and the categorical reduction of cultural diversity [3]. This trend, coupled with the invalidation of progressive and universal evolutionism, instigated a deep mistrust for evolutionary approaches to the understanding of cultural diversity [4]. Second, kinship thinking has seemingly always been intimidating to anthropological scholars, as Malinowski famously worded it, “the average anthropologist…had his doubts whether the effort needed to master the bastard algebra of kinship is really worthwhile” [5]. But the rejection of the cross-cultural study of kinship by a majority of anthropologists left the field predominantly to cognitive anthropologists and linguists, many of which advocated increasingly technical and algebraic lines of research, making the topic even less accessible [6].

**Criticism against cultural phylogenetic methods**

The main criticism of a cultural phylogenetic approach is the idea that unlike biological evolution, cultural evolution may not be treelike [7-10]. Stephen Jay Gould famously stated that the evolution of species is very tree-shaped because once lineages have diverged they are very unlikely to recombine [11]. On the other hand, he considered that recombination was rampant in cultural evolution, due to horizontal borrowing between cultures [11]. This proposed dichotomy turns out to be a false one, as horizontal transfer appears to be much more widespread in biological evolution than what has long been assumed [12]. Moreover, the basic vocabulary which is now used to construct language trees is relatively resistant to borrowing [12], and the most frequently used words in a language have been found to evolve much more slowly – up to one hundred times slower in rate - than those words which are used less often [13]. Additionally, Currie and colleagues used a simulation method to formally investigate at which rate horizontal transfer can come to affect the phylogenetic inference of language trees, and found that analyses were only compromised at a rate of borrowing which would seem unrealistic in natural conditions [14]. One can therefore conclude that as long as these linguistic factors are integrated into a phylogenetic study, there is no reason cultural evolution should be less treelike than biological evolution, a fact which has been systematically examined and confirmed using phylogenetic methods [15, 16]. Further than that, the methods offer the possibility of visualizing and quantifying the respective influences of horizontal and vertical transmission in cultural evolution, through the use of phylogenetic networks [15] and the calculation of phylogenetic signal present in data [17].

**Psychological evidence**

A recent psychological study has shown that all systems of kinship terminology found in the world are cognitively optimal trade-offs between complexity of the system and communicative cost [58]. Systems only become more demanding in terms of memory and communication if there is a necessary increase in their complexity. What we would like to understand, are the causes driving these surges in terminological complexity, forcing it to become more complicated in some societies than in others, as well as how these causal factors operate. A more detailed understanding of the evolution of kinship terminology would help the understanding of human systems of kinship organization overall, and how these interact with the structuring of cooperative behaviour, a question which is understood to be crucial in the study of human evolutionary success [19, 20].

**References**

1. Rivers WHR.  Kinship and Social Organisation. Routledge Revivals; Routledge; 1914.

2. Lowie RH. Relationship terms .EncyclopÆdia Britannica .14th edn. 19: 84–6; 1929

3. D’Andrade R. The sad story of Anthropology 1950-1999. Cross-Cultural Research 2000; 34: 219-232.

4. Colleran H, Mace R. Contrasts and conflicts in anthropology and archaeology: The evolutionary/interpretive dichotomy in human behavioural research. In Darwinian Archaeologies: A Discussion, E. Cochrane and A. Gardner, eds. Walnut Creek, CA: Leftcoast Press; 2011.

5. Malinowski B. Kinship. Man 1930; 30: 19-29.

6. Jordan F. A phylogenetic analysis of the evolution of Austronesian sibling terminologies. Human Biology 2011; 83(2): 297-321.

7. Bateman R, Goddard I, O'Grady R, Funk VA, Mooi R, Kress WJ, Cannell P, Armstrong DF, Bayard D, Blount BG, Callaghan CA. Speaking of forked tongues: the feasibility of reconciling human phylogeny and the history of language. Current Anthropology 1990; 31: 1-24.

8. Moore JH. Putting anthropology back together again: the ethnogenetic critique of cladistic theory. American Anthropologist 1994; 925-948.

9. Borgerhoff Mulder M. Using phylogenetically based comparative methods in anthropology: more questions than answers. Evolutionary Anthropology: Issues, News, and Reviews 2001; 10(3), 99-111.

10. Borgerhoff Mulder M, Nunn CL, Towner MC. Cultural macroevolution and the transmission of traits. Evolutionary Anthropology: Issues, News, and Reviews 2006; 15(2), 52-64.

11. Gould SJ. An Urchin in the Storm. Norton, New York; 1987.

12. Gray RD, Greenhill SJ, Ross RM. The pleasures and perils of Darwinizing culture (with phylogenies). Biol. Theory 2007; 2, 360–375.

13. Pagel M, Atkinson QD, Meade A. Frequency of word-use predicts rates of lexical evolution throughout Indo-European history. Nature 2007; 449(7163), 717-720.

14. Currie TE, Greenhill SJ, Mace R. Is horizontal transmission really a problem for phylogenetic comparative methods? A simulation study using continuous cultural traits. Phil. Trans. R. Soc. B 2010; 365: 3903–3912.

15. Bryant D, Filimon F, Gray RD. Untangling our past: languages, trees, splits and networks. In: The evolution of cultural diversity: a phylogenetic approach. Left Coast Press, CA; 2005. pp. 67-83.

16. Collard M, Shennan SJ, Tehrani JJ. Branching versus blending in macroscale cultural evolution: A comparative study. In Mapping Our Ancestors. NJ: Aldine Transaction, New Brunswick; 2006. pp.53-88.

17. Pagel M . The maximum likelihood approach to reconstructing ancestral character states of discrete characters on phylogenies. Systematic biology 1999; 48(3), 612-622.

18. Kemp C, Regier T Kinship categories across languages reflect general communicative principles. Science 2012; 336(6084): 1049-1054.

19. Hrdy S. Mothers and Others: The Evolutionary Origins of Mutual Understanding. Cambridge, MA: Harvard University Press; 2009.

20. Bowles S, Gintis H. A Cooperative Species: Human Reciprocity and its Evolution. Princeton University Press, Oxford; 2011.
