# Supplementary material for: Correction: A Phylogenetic Comparative Study of Bantu Kinship Terminology Finds Limited Support for Its Co-Evolution with Social Organisation
Source: PLoS One. 2016 May 3;11(5):e0155170. doi: 10.1371/journal.pone.0155170 (PMC4854460; doi:10.1371/journal.pone.0155170)
Supplement: S1 Fig — The six typologies created by Murdock, and how kin is referred to in relation to ego in each system. (PDF) [file pone.0155170.s001.pdf]

Hawaiian

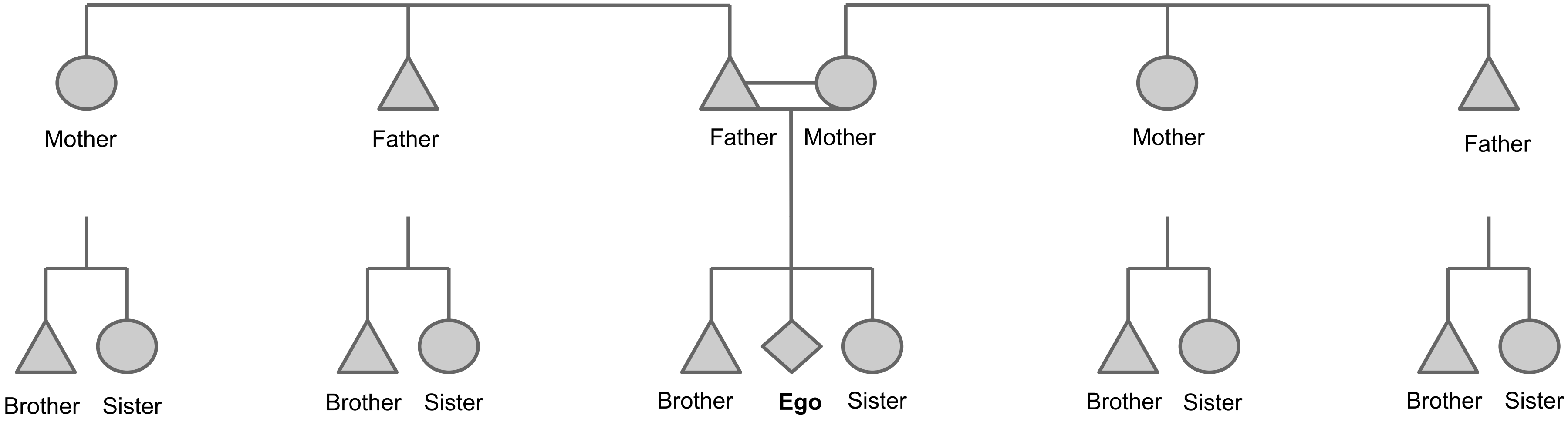

The Hawaiian terminological system is the most classificatory system, distinguishing only by sex and generation.

Eskimo

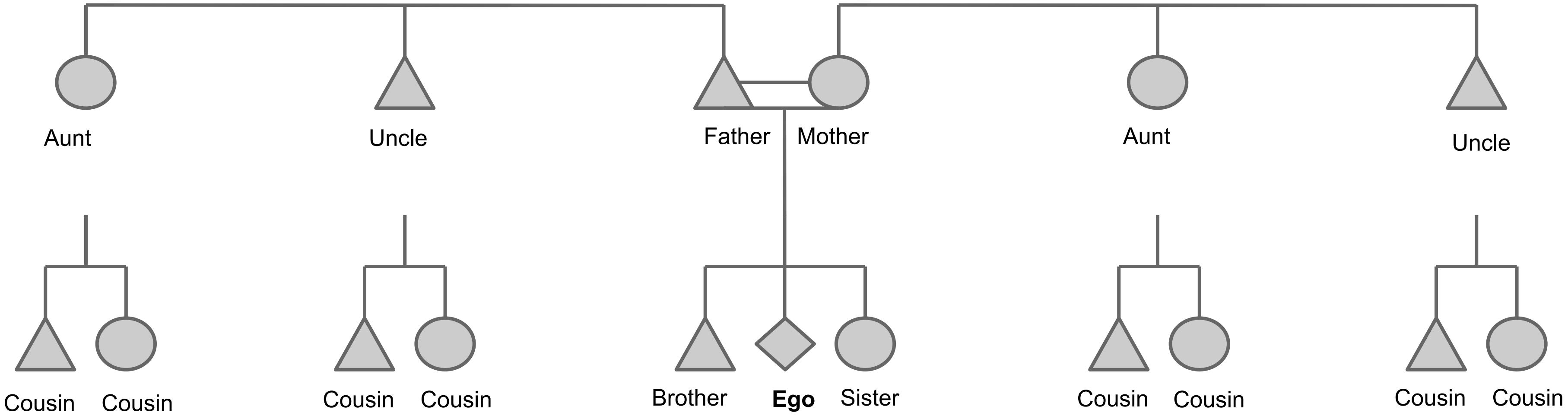

The Eskimo terminological system emphasizes the nuclear family, distinguishing between collateral and lineal relatives.

Iroquois

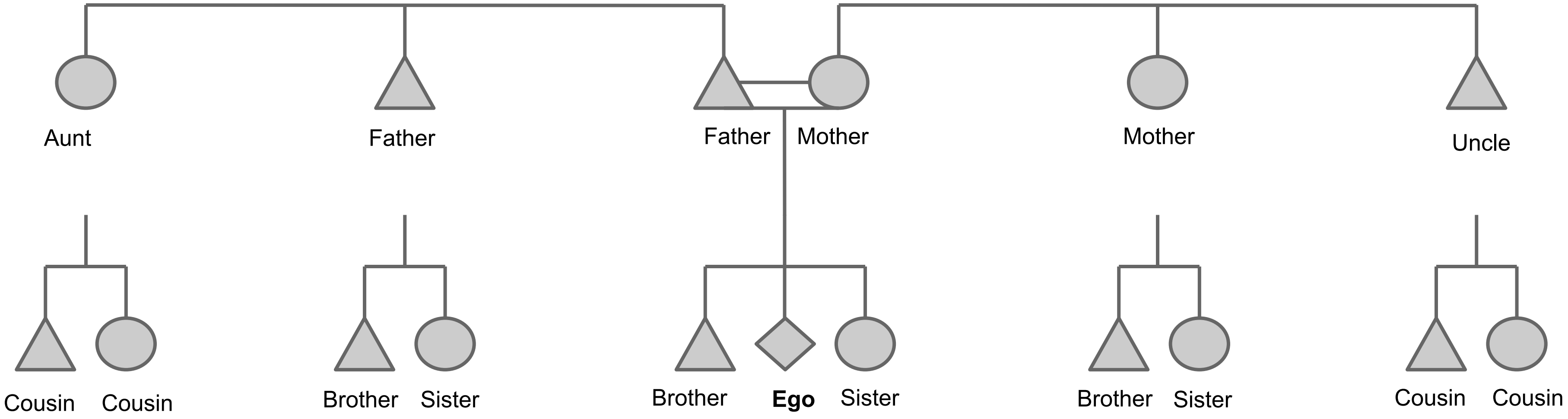

The Iroquois terminological system emphasizes bifurcation, and distinguishes between parallel and cross cousins.

Omaha

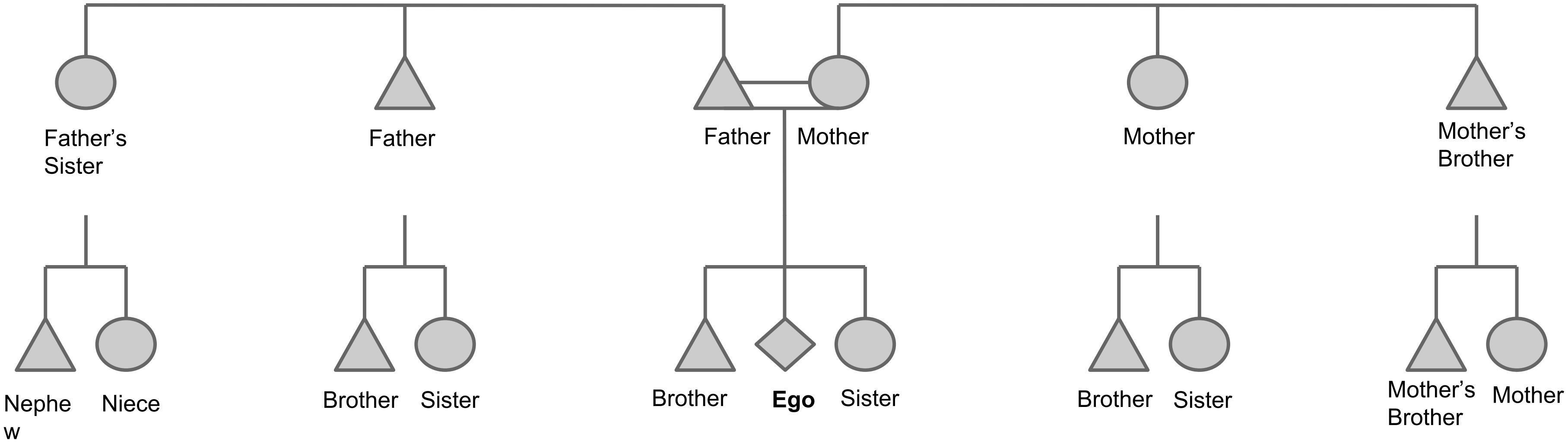

The Omaha terminological system emphasizes paternal kin by using more descriptive terms for these relatives, and ignoring the generational distinction for maternal cross-cousins, making maternal kin terms more classificatory.

Crow

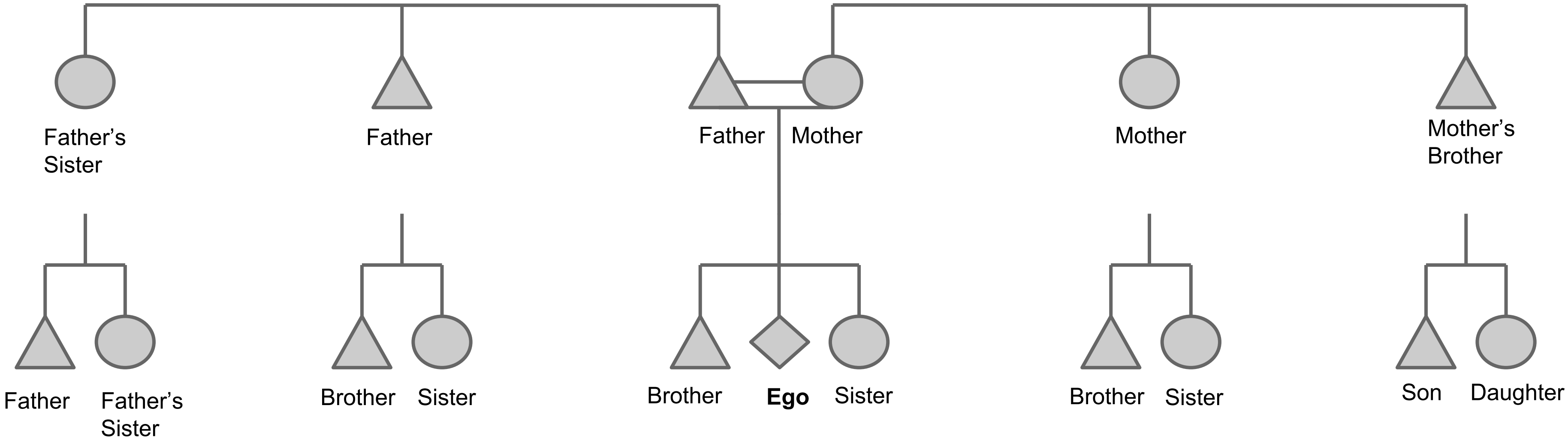

The Crow terminological system emphasizes maternal kin by using more descriptive terms for these relatives, and ignoring the generational distinction for paternal cross-cousins, making paternal kin terms more classificatory.

Sudanese

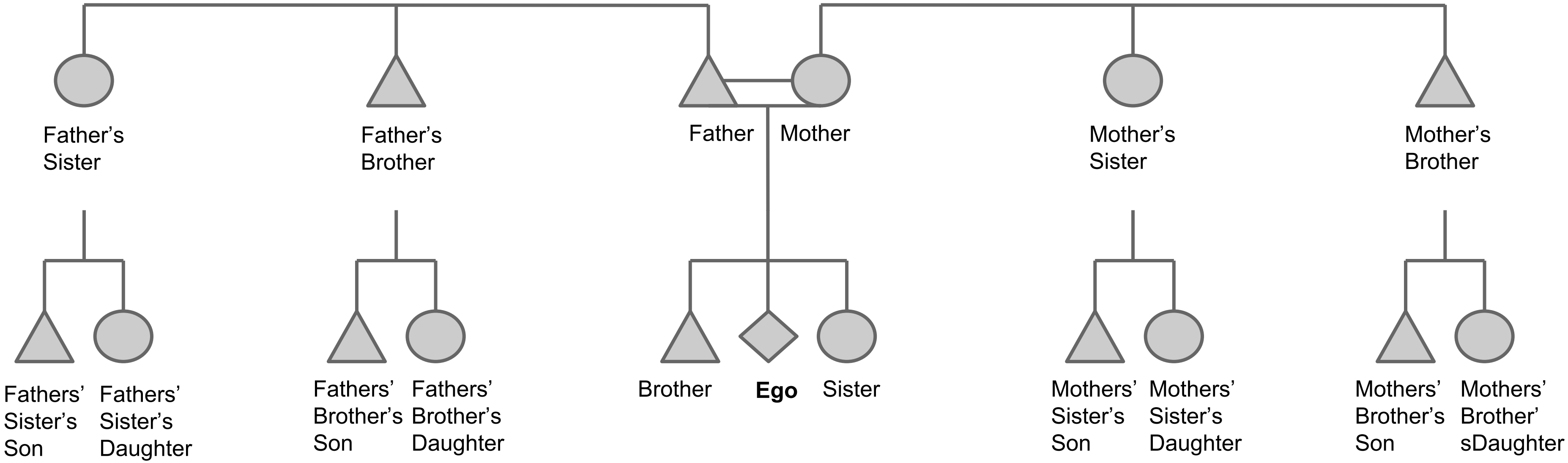

The Sudanese terminological system is the most descriptive, distinguishing between most types of kin.
